# ADAP2 Is an Interferon Stimulated Gene That Restricts RNA Virus Entry

**DOI:** 10.1371/journal.ppat.1005150

**Published:** 2015-09-15

**Authors:** Qian Shu, Nicholas J. Lennemann, Saumendra N. Sarkar, Yoel Sadovsky, Carolyn B. Coyne

**Affiliations:** 1 Tsinghua University School of Medicine, Beijing, China; 2 Department of Microbiology and Molecular Genetics, University of Pittsburgh, Pittsburgh, Pennsylvania, United States of America; 3 University of Pittsburgh Cancer Institute, University of Pittsburgh, Pittsburgh, Pennsylvania, United States of America; 4 Magee-Womens Research Institute, Department of Obstetrics, Gynecology, and Reproductive Science, University of Pittsburgh, Pittsburgh, Pennsylvania, United States of America; University of Washington, UNITED STATES

## Abstract

Interferon stimulated genes (ISGs) target viruses at various stages of their infectious life cycles, including at the earliest stage of viral entry. Here we identify ArfGAP with dual pleckstrin homology (PH) domains 2 (ADAP2) as a gene upregulated by type I IFN treatment in a STAT1-dependent manner. ADAP2 functions as a GTPase-activating protein (GAP) for Arf6 and binds to phosphatidylinositol 3,4,5-trisphosphate (PI(3,4,5)P_3_) and PI(3,4)P_2_. We show that overexpression of ADAP2 suppresses dengue virus (DENV) and vesicular stomatitis virus (VSV) infection in an Arf6 GAP activity-dependent manner, while exerting no effect on coxsackievirus B (CVB) or Sendai virus (SeV) replication. We further show that ADAP2 expression induces macropinocytosis and that ADAP2 strongly associates with actin-enriched membrane ruffles and with Rab8a- and LAMP1-, but not EEA1- or Rab7-, positive vesicles. Utilizing two techniques—light-sensitive neutral red (NR)-containing DENV and fluorescence assays for virus internalization—we show that ADAP2 primarily restricts DENV infection at the stage of virion entry and/or intracellular trafficking and that incoming DENV and VSV particles associate with ADAP2 during their entry. Taken together, this study identifies ADAP2 as an ISG that exerts antiviral effects against RNA viruses by altering Arf6-mediated trafficking to disrupt viral entry.

## Author Summary

The induction of antiviral innate immune signaling is a primary defense strategy employed by host cells to restrict virus infections. This system is triggered by the presence of ‘non-self’ components such as viral nucleic acids and culminates in the induction of type I interferons (IFNs). Type I IFNs themselves possess no direct antiviral activity and instead exert their potent antiviral effects via the induction of hundreds of interferon-stimulated genes (ISGs) that directly antagonize viruses at a variety of steps in their infectious cycles. Here we identify ArfGAP with dual pleckstrin homology (PH) domains 2 (ADAP2) as an ISG whose expression restricts the replication of dengue virus (DENV), which infects as many as 400 million people worldwide annually, at the stage of virion entry and/or trafficking. Our study thus not only identifies ADAP2 as a previously uncharacterized ISG, but also points to its role in the inhibition of DENV replication at the earliest stages of the DENV life cycle.

## Introduction

The induction of innate immune signaling is critical for host defense against viral infections, and is most commonly initiated by the detection of foreign nucleic acids as non-self. Once this system is activated, host cells orchestrate an array of signaling pathways that culminate in the induction of type I interferons (IFNs), which include IFNα and IFNβ. Type I IFNs themselves possess no antiviral activity and instead exert their potent antiviral effects by the induction of hundreds of interferon-stimulated genes (ISGs) that can be induced by secreted IFNs in either an autocrine or paracrine manner. ISGs can function in a pan-viral manner or can target specific virus species and/or families [1]. Although the mechanisms by which several ISGs function to suppress viral infections have been well documented, the functions of many ISGs remain largely undefined.

ISGs function to restrict viral replication at various stages of the viral life cycle, with some ISGs targeting the earliest event associated with infection—viral entry into the host cell. Some of the best-characterized ISGs that target viral entry belong to the interferon-inducible transmembrane protein (IFITM) family, which includes IFITM1, IFITM2, IFITM3, and IFITM5 (reviewed in [2, 3]). IFITM family members exert broad antiviral effects against a diverse range of viruses including influenza A virus [4], HIV [5], dengue virus (DENV) [4], and Ebola virus [6]. Although expressed at basal levels in many cell types, IFITM family members are induced by type I IFNs and may inhibit viral entry by direct alterations of cellular cholesterol homeostasis [7], by alterations in the fusion between vesicular compartments that may favor a non-infectious entry pathway [8], and/or by preventing fusion between viral and host-derived membranes while still permitting hemifusion [8, 9]. The targeting of viruses at the earliest stages of their infectious life cycles serves as a potent step at which ISGs can antagonize viral infections.

Viruses enter host cells through a variety of mechanisms. In some cases, viruses enter cells through similar means across multiple cell types, whereas in others, they utilize cell type-specific mechanisms for their entry. For example, vesicular stomatitis virus (VSV) exclusively utilizes a clathrin-mediated pathway for its entry [10, 11], whereas the enterovirus coxsackievirus B (CVB) enters via a dynamin II GTPase-independent pathway in polarized intestinal cells [12], but enters nonpolarized epithelial cells and polarized epithelial cells through a dynamin II-mediated pathway [13, 14]. Some viruses, such as Sendai virus (SeV) fuse directly at the host cell plasma membrane [15]. In the case of viruses that infect across diverse species, such as DENV that gains entry into both mosquito and human cells during its life cycle, viruses also exhibit species-specific entry mechanisms. In mosquito cells, DENV has been proposed to both enter via a clathrin-mediated pathway that requires delivery of incoming virions to a low pH endolysosomal compartment for fusion [16], and possibly also by direct fusion at the plasma membrane [17]. In human cells, data support an uptake pathway for DENV that requires both clathrin coated pits and delivery of incoming virions to late endosomes [16, 18]. Cellular factors that restrict virus entry may thus exhibit virus and cell-type specificity depending on the mechanism by which the virus gains entry into the host cell and/or mediates its uncoating and/or fusion.

The process of endocytosis is tightly regulated by diverse cellular factors that specifically control the complex stages of the endocytic process. Many of these factors are GTP-binding proteins whose activity is regulated by both guanine nucleotide exchange factors (GEFs) and GTPase-activating proteins (GAPs). Members of the ADP-ribosylation factor (Arf) family of Ras-related proteins are expressed in all eukaryotic cells and regulate both secretory pathway trafficking (as is the case for Arf1) as well as cortical actin rearrangements and endocytosis (as is the case for Arf6). Arf6 influences cell migration, polarization, endocytosis, and endosomal trafficking via its direct impact on both regulators of the actin cytoskeleton [19, 20] and the lipid membrane [21, 22]. Thus, the expression of regulators of Arf6 activity, such as Arf6 GEFs and GAPs, can induce pronounced effects on a multitude of cellular events converging on uptake from the plasma membrane and/or endosomal trafficking.

To identify additional ISGs that might directly impact virus entry and/or intracellular trafficking, we performed microarray analyses in control and signal transducer and activator of transcription (STAT)-1 signaling deficient cells exposed to purified IFNβ. Using this approach, we identified ArfGAP with dual pleckstrin homology (PH) domains 2 (ADAP2) as a gene upregulated by IFNβ exposure in a STAT1-dependent manner. ADAP2 (also known as centaurin-α_2_, CENTA2) is a phosphatidylinositol 3,4,5-trisphosphate (PI(3,4,5)P_3_) and PI(3,4)P_2_ binding protein whose expression alters Arf6 membrane localization [23–25]. However, ADAP2 has not been fully characterized and relatively little is known regarding its role in endocytosis or endosomal trafficking. Here we show that expression of ADAP2 suppresses DENV and VSV infection in an Arf6 GAP activity-dependent manner, while exerting no effect on CVB or SeV replication. We further show that expression of ADAP2 induces pronounced effects on the actin cytoskeleton and that it directly associates with actin-enriched membrane ruffles, macropinosomes, and lysosomes. Utilizing two techniques—a light-sensitive neutral red (NR)-containing DENV and fluorescence assays for virus internalization-—we show that ADAP2 primarily restricts DENV and VSV infection at the stage of virion entry or trafficking. Taken together, this study identifies an ISG that exerts its effects on DENV replication by altering Arf6-mediated trafficking to disrupt viral entry/trafficking.

## Results

### ADAP2 is induced by IFNβ treatment in a STAT1-dependent manner

**Fig 1:**
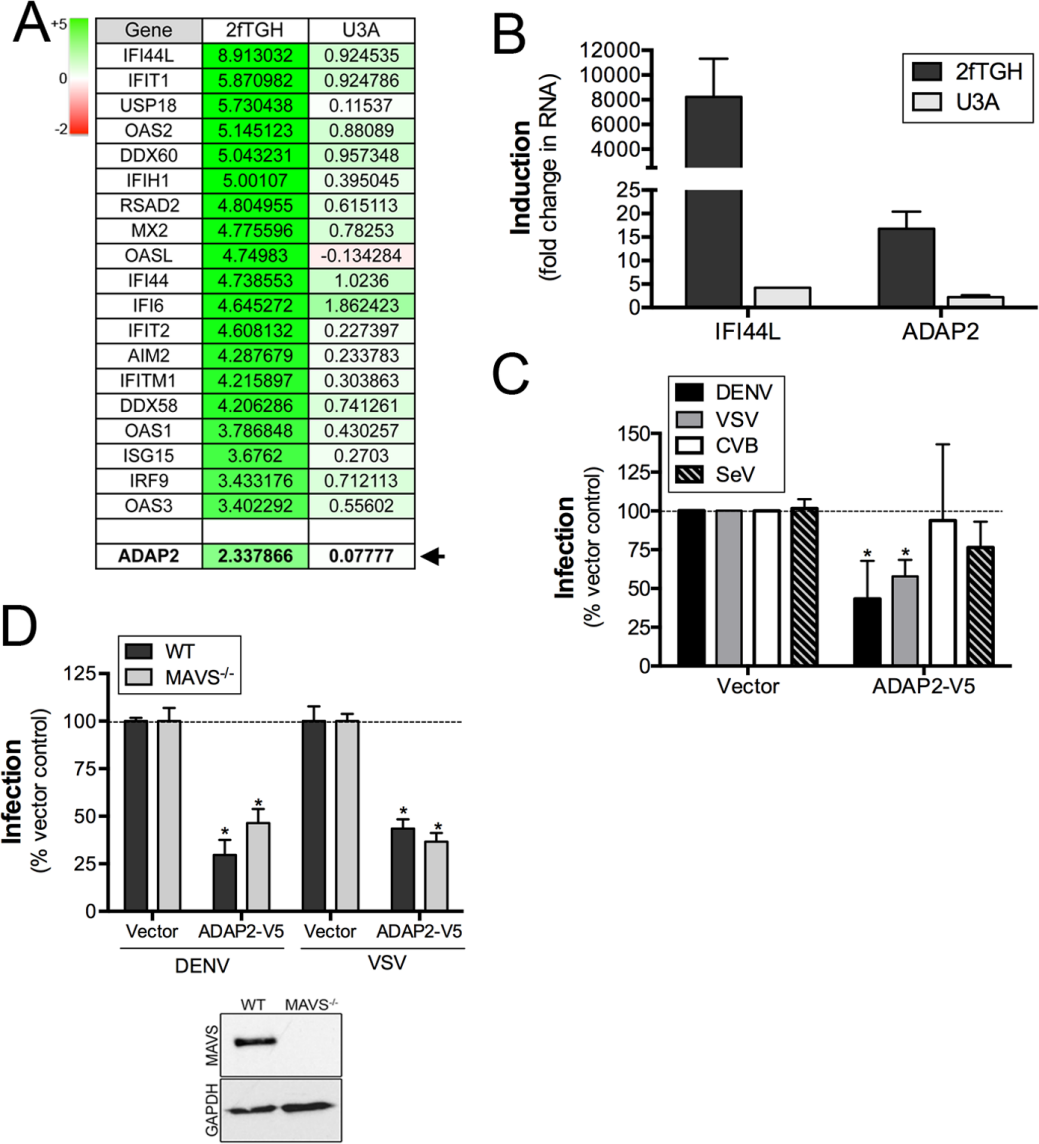
ADAP2 is induced by IFNβ treatment in a STAT1-dependent manner and restricts DENV and VSV infection. **(A),** Table of select genes enhanced by IFNβ exposure of control (2fTGH), but not STAT1 signaling deficient (U3A) HT1080 cells. Shown are log scale expression of the indicated genes following IFNβ relative to mock-treated controls. Green shading indicates the magnitude of unregulation whereas red shading indicates the magnitude of downregulation (top left). ADAP2 is shown in bold and is denoted by an arrow. **(B)**, RT-qPCR analysis for IFI44L and ADAP2 in 2fTGH (dark grey) or U3A (light grey) HT1080 cells treated with 100U of purified IFNβ for 24hrs. Expression is shown as fold change relative to untreated cells. **(C),** Infection of DENV (1 FFU/cell for 24hrs, dark grey), VSV (0.2 PFU/cell for 8hrs, light grey), CVB (0.2 PFU/cell for 8hrs, white), or SeV (100HAU/mL for 24hr, hatched) as assessed by RT-qPCR in 293T cells transfected with V5-fused ADAP2 or vector control. Data are normalized to vector control-infected cells. **(D),** DENV (1 FFU/cell for 24hrs) and VSV (0.2 PFU/cell for 8hrs) infection as assessed by RT-qPCR in wild-type (WT) or MAVS-deficient (MAVS^-/-^) 293T cells transfected with vector control or V5-fused ADAP2. Data are shown as percent infection of vector controls. Inset, immunoblot for MAVS (top panel) or GAPDH (bottom panel) in WT or MAVS^-/-^ cells. Data in (C) and (D) are shown as mean ± standard deviation and are from three independent experiments. Data in (B) are representative of three independent experiments. In (C) and (D), *p<0.05.

To identify ISGs that might impact events associated with virus entry, we performed microarray analysis in control (2fTGH) human fibrosarcoma HT1080 cells or cells lacking functional STAT1 (U3A) treated with purified IFNβ. As expected, we found that many known ISGs, such as interferon-induced protein 44-like (IFI44L), members of the interferon-induced protein with tetratricopeptide repeats (IFIT) family, radical S-adenosyl methionine domain containing 2 (RSAD2), and members of the 2',5'-Oligoadenylate synthetase (OAS) family were upregulated by IFNβ treatment in a STAT1-mediated manner (Fig 1A and S1 Table). In addition, we noted that the expression of ADAP2, which contains Arf6 GAP activity and associates with actin cytoskeletal rearrangements, was also enhanced by IFNβ treatment in a STAT1-depdendent manner both by microarray (Fig 1A) and in follow up studies by RT-qPCR (Fig 1B). Given the association of Arf6 with events that might impact viral entry, we chose to characterize both the antiviral and cell biological properties of ADAP2.

### Expression of ADAP2 restricts DENV and VSV replication

To determine whether ADAP2 exerts antiviral activity, we assessed the effects of its ectopic expression on the replication of DENV, VSV, CVB and SeV. We found that ADAP2 expression partially restricted DENV and VSV infection, but had no consistent effect on CVB or SeV infection (Fig 1C). Expression of ADAP2 was confirmed by RT-qPCR (S1A Fig). We also found that ADAP2 expression restricted DENV infection across a variety of multiplicity of infections (MOIs) (S1B Fig). In addition, we found similar antiviral effects when EGFP-fused ADAP2 was ectopically expressed (S1C and S1D Fig). Although overexpression of ADAP2 restricted DENV and VSV infection, we found that silencing of ADAP2 led to a mild increase in DENV infection (S1H Fig). We attribute this result to the hundreds of other ISGs that are induced in response to viral infection and work in parallel to suppress viral infections.

To exclude any impact of antiviral signaling on the restriction of DENV replication by ADAP2, we next assessed the impact of ADAP2 overexpression on DENV and VSV replication in 293T cells depleted of the RIG-I-like receptor (RLR) adaptor mitochondrial antiviral signaling (MAVS) by CRISPR Cas9 genome editing. We found that expression of ADAP2 restricted DENV and VSV replication in both control cells and in cells lacking the expression of MAVS (Fig 1D), supporting a RIG-I-like receptor (RLR)-independent pathway in the antiviral effects of ADAP2. Expression of ADAP2 was confirmed by RT-qPCR (S1E Fig). In addition, expression of ADAP2 has no impact on the induction of ISGs during DENV infection and instead reduced the induction given the inhibition of viral replication (S1F and S1G Fig). Taken together, these data implicate a non-IFN-based mechanism of antiviral activity of ADAP2.

### ADAP2 associates with actin-enriched membrane ruffles

**Fig 2:**
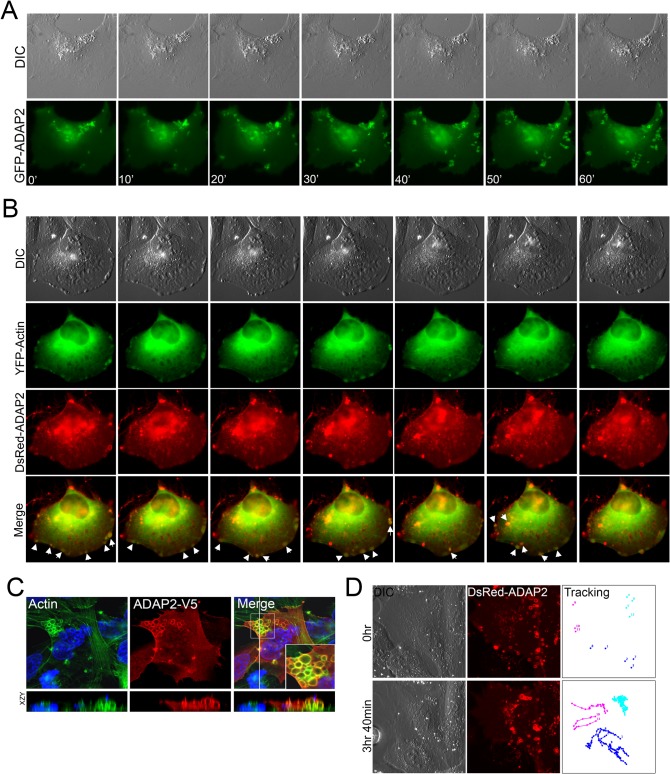
ADAP2 associates with membrane ruffles and actin. **(A),** Selected frames (captured every 10min) from time-lapse movies of U2OS cells expressing GFP-ADAP2. See S1 Movie for complete movie. At top, differential interference contrast (DIC). **(B),** Selected frames (captured every 10min) from time-lapse movies of U2OS cells expressing YFP-actin and DsRed-ADAP2. See S2 Movie for complete movie. At top, differential interference contrast (DIC). Newly formed actin-enriched membrane ruffles associated with DsRed-ADAP2 are denoted by white arrows. **(C)**, Immunofluorescence microscopy for actin (in green) in U2OS cells transfected with ADAP2-V5. Cells were fixed and immunostained ~48hrs post-transfection. Areas of colocalization appear as yellow. White box denotes zoomed image at bottom right of merged image. Xy image shown at top and xzy cross-section shown at bottom (cross-section corresponds to the white line shown in the merged panel). **(D),** Single particle tracking from time-lapse movies of U2OS cells expressing DsRed-ADAP2 over the period of ~3.5hrs. Three independent vesicles were tracked and are shown in light blue, dark blue, or pink. At top, initial image acquired prior to particle tracking (0hr) and at bottom, image acquired at the conclusion of image acquisition. At left, DIC image. See S3 Movie for complete movie.

Relatively little is known regarding the localization and trafficking of ADAP2. Therefore, we defined the localization of ADAP2 using real-time fluorescence microscopy in cells transfected with GFP-fused ADAP2 over the course of 48hrs post-transfection. Utilizing this approach, we found that expression of ADAP2 induced pronounced membrane ruffling and that it associated with both membrane ruffles and the vesicles that internalized from these ruffles (Fig 2A, S1 Movie). Unfortunately, immunostaining with all commercially available ADAP2 antibodies was unsuccessful, thus we proceeded to characterize the cell biological localization of ADAP2 with ectopically expressed fusion proteins.

To determine whether the ruffles induced by ADAP2 expression were actin-enriched, we also performed extended real-time fluorescence microscopy in cells transfected with YFP-fused actin and DsRed-fused ADAP2. We found that DsRed-ADAP2 was associated with YFP-actin both at membrane ruffles and with vesicles internalizing from these ruffles (Fig 2B, S2 Movie). In addition, we noted that in approximately 5–10% cells, ADAP2 overexpression induced the formation of very large actin-coated vacuoles that accumulated in large clusters (Fig 2C).

We next performed single vesicle-tracking analyses in cells expressing ADAP2 over an extended period (~4hrs) to determine the pattern of movement exhibited by ADAP2-containing vesicles (S3 Movie). We found that ADAP2-containing vesicles exhibited three distinct patterns of movement—(1) vesicles originating at the cell periphery/membrane ruffles that quickly internalized to the perinuclear region (Fig 2D, pink tracings), (2) preformed vesicles that exhibited free movement within the cell (Fig 2D, dark blue tracings), and (3) vesicles that were largely stationary and exhibited restricted movements (Fig 2D, light blue tracings). Taken together, these data implicate ADAP2 in the enhancement of actin-enriched membrane ruffling.

### ADAP2 associates with macropinosomes

**Fig 3:**
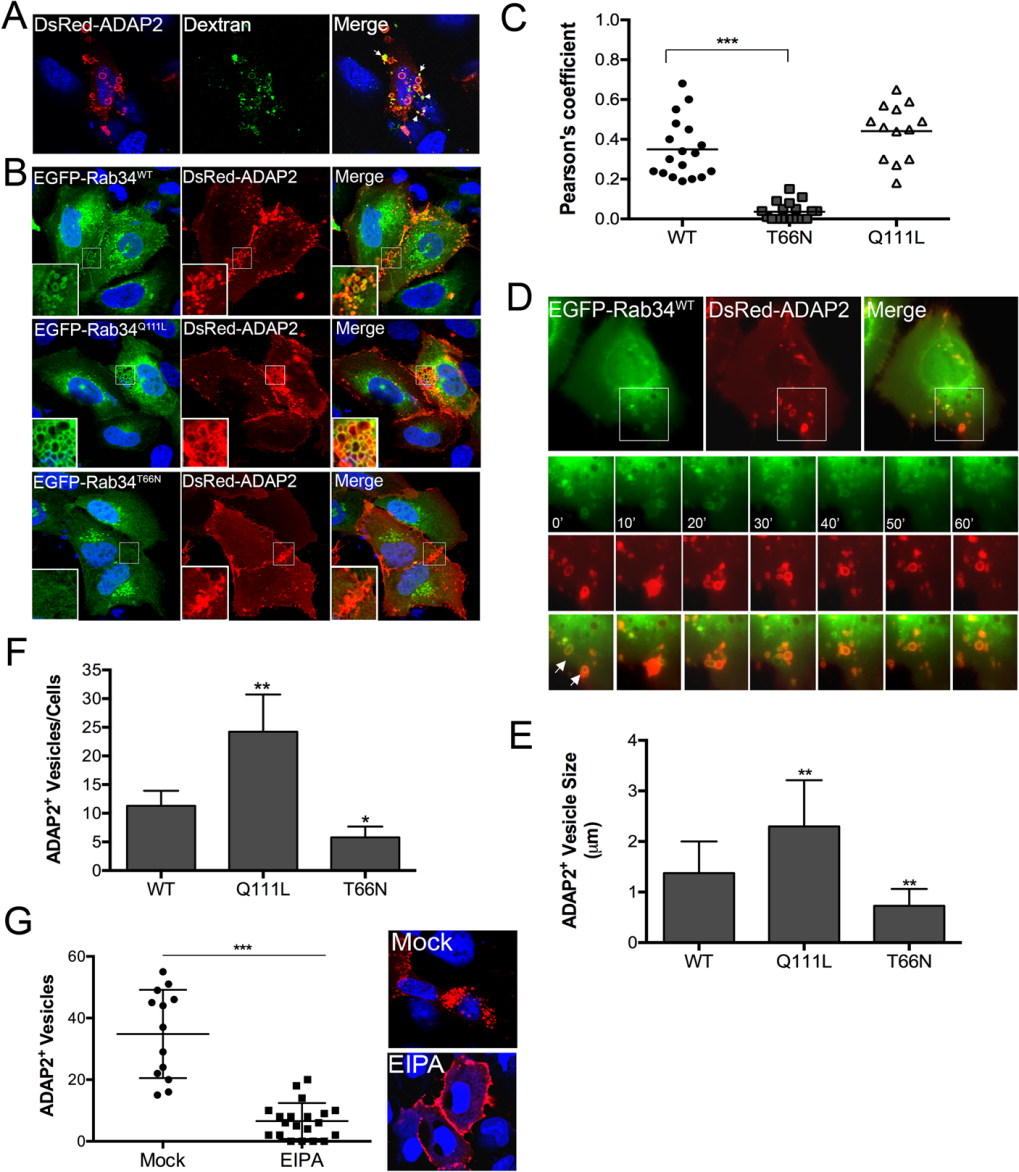
ADAP2 induces macropinocytosis and associates with macropinosomes. **(A),** Uptake of fluorescently-labelled 70kD dextran (green) in U2OS cells transfected with DsRed-ADAP2. Approximately 48hrs following transfection, U2OS cells were incubated with dextran (0.1 mg/ml) for 45 min. White areas denote areas of colocalization. **(B),** Confocal micrographs of U2OS cells transfected with wild-type (WT), constitutively-active (Q111L), or dominant-negative (T66N) EGFP-Rab34 and DsRed-ADAP2. Areas of colocalization appear as yellow. White boxes denote zoomed images shown at bottom left. **(C)** Pearson correlation coefficient to assess the colocalization between ADAP2 and wild-type, T66N, or Q111L Rab34. Each point represents a unique cell that coexpressed both constructs. Line indicates the mean. **(D),** Selected frames (captured every 10min) from time-lapse movies of U2OS cells expressing EGFP-Rab34^WT^ and DsRed-ADAP2. See S4 Movie for complete movie. Newly formed Rab34-enriched membrane ruffles associated with DsRed-ADAP2 are denoted by white arrows. **(E, F),** Quantification of the number of ADAP2+ vesicles (E) or the sizes of ADAP2^+^ vesicles (F) in U2OS cells transfected with the indicated Rab34 constructs. **(G),** U2OS cells transfected with DsRed-ADAP2 were treated with EIPA (102 μM) for 1hr ~48hrs post-transfection. Cells were fixed and the number of ADAP2+ vesicles calculated (graph at left). At right, representative confocal micrograph of mock- or EIPA-treated cells. DAPI-stained nuclei are shown in blue and DsRed-ADAP2 is in red. Data in (E-F) are shown as mean ± standard deviation and are from at least 20 independent vesicles from at least three independent fields. In (D) and (E), *p<0.05 and **p<0.01.

A hallmark of internalized macropinosomes is their association with actin. Given that we found that ADAP2 associated with actin-enriched membrane ruffles and internalized vesicles, we next assessed whether these vesicles were macropinosomes. To do this, we analyzed the localization of ADAP2 with fluorescently-labeled dextran, which serves as a marker for fluid-phase uptake. We found that expression of ADAP2 not only enhanced the extent of dextran uptake, but that ADAP2-containing cytoplasmic vesicles were enriched in dextran (Fig 3A).

Next, we determined the impact of expression of wild-type or constitutively-active (Q111L) and dominant-negative (T66N) mutants of Rab34, a GTPase that has been specifically associated with macropinocytosis [26], on the ability of ADAP2 to induce vesicles. We found that ADAP2 associated with Rab34-containing vesicles when both wild-type (Fig 3B and 3C, S4 Movie) and Q111L mutant (Fig 3B and 3C) were expressed. In addition, we found that expression of Rab34 Q111L enhanced the numbers and sizes of ADAP2-containing vesicles whereas expression of T66N Rab34 significantly reduced these vesicles and induced the accumulation of ADAP2 at cell-cell contacts and membrane ruffles (Fig 3B–3F).

Further supporting a role for macropinocytosis in the formation of ADAP2-enriched vesicles, we found that treatment of cells with 5-(N-ethyl-N-isopropyl)-Amiloride (EIPA), an inhibitor of macropinoctyosis [27], led to the accumulation of ADAP2 at the cell periphery and inhibited the formation of ADAP2-enriched vesicles (Fig 3G). Taken together, these data support a role for ADAP2 in the induction of macropinocytosis, or a macropinocytosis-like process, in a Rab34-mediated manner.

### ADAP2 associates with Rab8a-positive recycling endosomes

**Fig 4:**
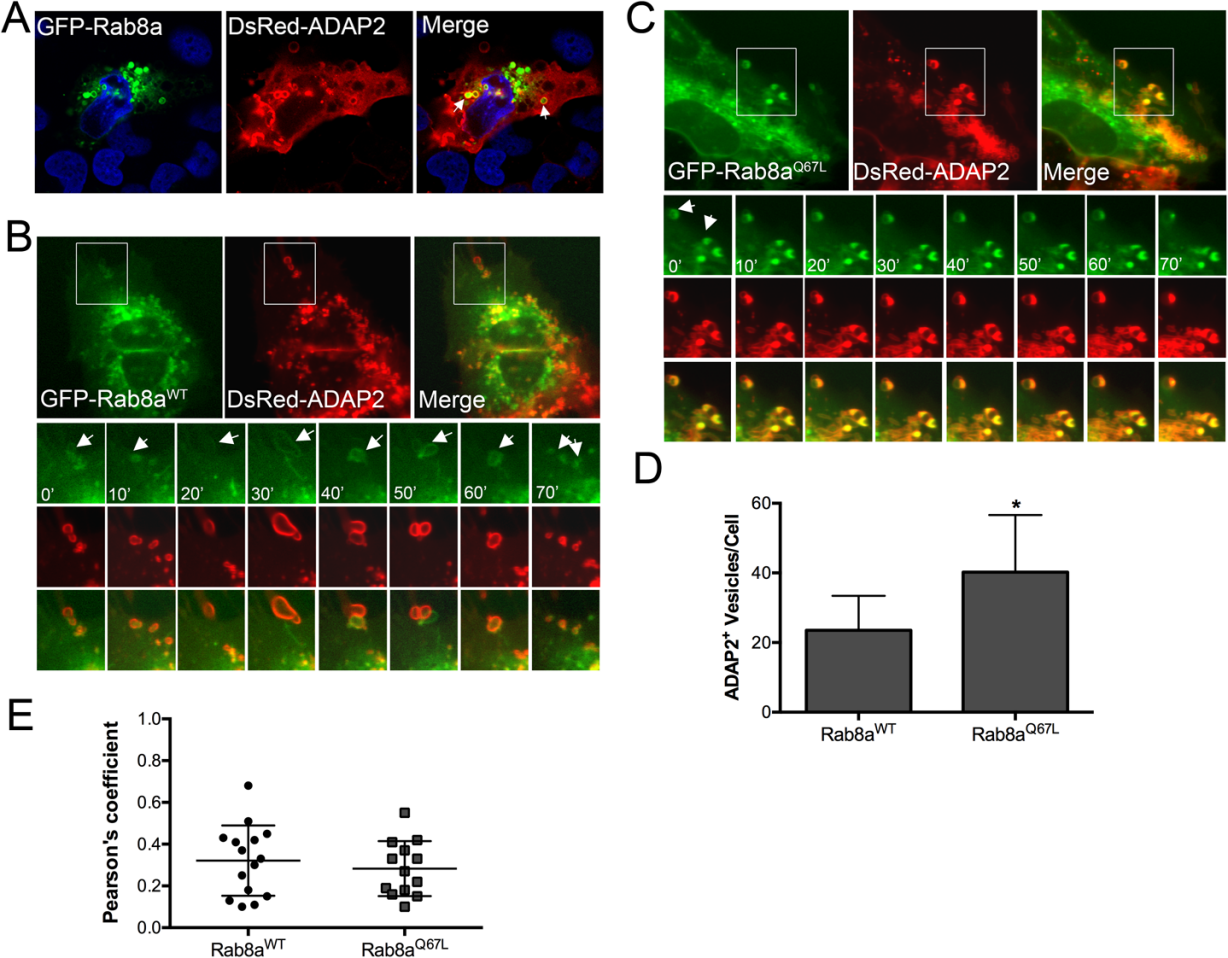
ADAP2 associates with Rab8a-positive membrane ruffles and recycling endosomes. **(A),** Confocal micrographs of U2OS cells transfected with wild-type GFP-Rab8a and DsRed-ADAP2. Areas of colocalization appear as yellow. DAPI-stained nuclei are shown in blue. **(B, C),** Selected frames (captured every 10min) from time-lapse movies of U2OS cells expressing GFP-Rab8a^WT^ (B) or constitutively-active GFP-Rab8a^Q67L^ and DsRed-ADAP2. See S5 and S6 Movies for complete movies. **(D),** Quantification of the number of ADAP2+ vesicles in cells expressing either wild-type or Q67L Rab8a. **(E),** Pearson correlation coefficient to assess the colocalization between ADAP2 and wild-type, or Q67L Rab8a. Each point represents a unique cell that coexpressed both constructs. Line indicates the mean. Data in (D) are shown as mean ± standard deviation and are from at least 20 independent vesicles from at least three independent fields (*p<0.05).

Rab8a GTPase localizes to membrane ruffles and macropinosomes as well as to tubular recycling endosomes that lead to membrane delivery back to the cell membrane [28, 29]. Given that Rab8a localizes to macropinosomes, and that Arf6 functions upstream of Rab8a in this pathway [28], we next determined whether ADAP2 associates with Rab8a. We found that Rab8 and ADAP2 exhibited significant colocalization in intracellular vesicles both in fixed (Fig 4A) and living (Fig 4B and 4E, S5 Movie) cells. In addition, we found that expression of a constitutively active mutant of Rab8a (Q67L) also highly associated with ADAP2-containing vesicles (Fig 4C, S6 Movie) and enhanced the numbers of total ADAP2 vesicles (Fig 4D). As we detected ADAP2 at membrane ruffles and within intracellular cytoplasmic vesicles, these data suggest that ADAP2 localizes to Rab8a-positive recycling endosomes in addition to macropinosomes.

### ADAP2 associates with LAMP1-positive lysosomes

**Fig 5:**
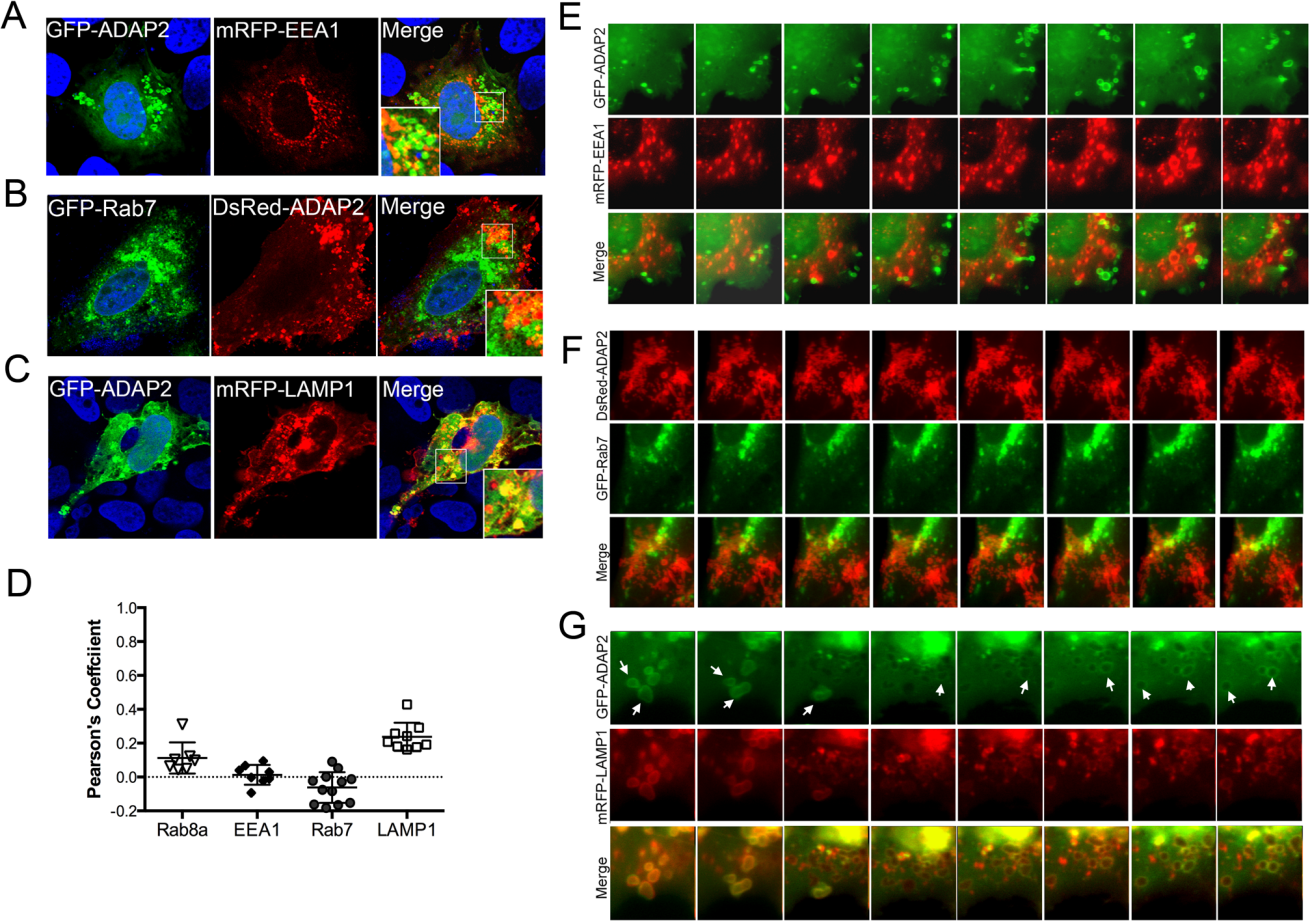
ADAP2 associates with LAMP1-positive lysosomes, but not early or late endosomes. **(A-C),** Confocal micrographs of U2OS cells transfected with mRFP-EEA1 (A), GFP-Rab7 (B), or mRFP-LAMP1 (C) and either GFP-ADAP2 (A, C) or DsRed-ADAP2 (B). Areas of colocalization appear as yellow. DAPI-stained nuclei are shown in blue. White boxes denotes zoomed areas shown in bottom left (A) or right (B, C) corners. **(D),** Quantification of the extent of colocalization (as assessed by Pearson’s correlation coefficients) in cells transfected with ADAP2 and the indicated endosomal or lysosomal markers. Data are shown as individual Pearson’s correlation coefficients quantified from individual cells. Coefficients >0 indicated positive colocalization. **(E-G),** Selected frames (captured every 10min) from time-lapse movies of U2OS cells expressing mRFP-EEA1 (E), GFP-Rab7 (F), or mRFP-LAMP1 (G) and either GFP-ADAP2 (E, G) or DsRed-ADAP2 (F). See S7–S9 Movies for complete movies. In G, the newly formed ADAP2-positive vesicles are highly associated with LAMP1 and are denoted by white arrows.

Once internalized, conventional macropinosomes often fuse with one another, but generally exhibit little fusion with endosomes or lysosomes [30]. However, in some circumstances, internalized macropinosomes fuse directly with tubular lysosomes following their maturation [31]. To define the maturation process of ADAP2-containing macropinosomes, we assessed the localization of GFP-ADAP2 with markers of early endosomes (early endosome antigen-1 (EEA1)), late endosomes (Rab7), and lysosomes (LAMP1). We found that whereas ADAP2-containing vesicles were excluded from EEA1- and Rab7-containing vesicles, they exhibited a strong association with LAMP1-containing vesicles in both fixed (Fig 5A–5D) and living (Fig 5E–5G, S7–S9 Movies) cells. These data indicate that upon their internalization, ADAP2-containing vesicles strongly associate with LAMP1-positive lysosomes.

### The Arf6 GAP activity of ADAP2 is required for its induction of macropinocytosis

**Fig 6:**
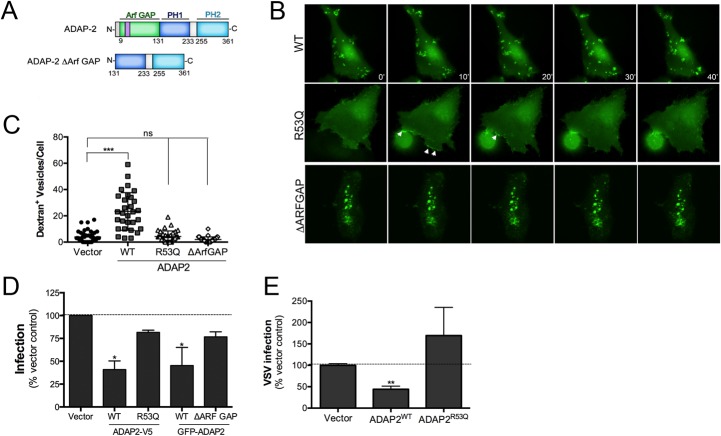
The Arf6 GAP activity of ADAP2 is required for its induction of macropinocytosis and antiviral effects. **(A),** Schematic of full-length ADAP2 or mutants lacking either the entire Arf6 GAP domain (ADAP2 ΔArf GAP) or the second PH domain (ADAP2 ΔPH2). **(B),** Selected frames (captured every 10min) from time-lapse movies of U2OS cells expressing wild-type, Arf6 GAP inactive (R53Q), or ΔArf GAP GFP-ADAP2. See S10–S12 Movies for complete movies. **(C),** Quantification of the extent of dextran uptake in U2OS cells transfected with vector control or EGFP-fused wild-type, R53Q, or ΔARFGAP ADAP2. Following transfection with the indicated constructs for ~48hrs, cells were incubated with Alexa Fluor 594 dextran (0.1 mg/ml) for 45 min, then fixed, and images captured. The numbers of dextran+ vesicles in each condition was then measured. Each point represents a unique cell that expressed the indicated constructs (>20 total cells from at least three independent experiments were calculated). ***p<0.001, ns (not significant). **(D)**, DENV infection (1 FFU/cell for 24hrs) as assessed by RT-qPCR in 293T cells transfected with vector control or either wild-type (WT) or R53Q V5-fused or GFP-fused ADAP2. **(E)**, VSV infection (0.2 PFU/cell for 8hrs) as assessed by RT-qPCR in 293T cells transfected with vector control or either wild-type (WT) or R53Q V5-fused ADAP2. Data in (D) and (E) are shown as mean ± standard deviation (*p<0.05, **p<0.01).

ADAP2 contains an Arf6 GAP domain and two PH domains. We next determined whether the Arf6 GAP activity of ADAP2 was required for its induction of macropinocytosis. To do this, we constructed ADAP2 mutants in which the entire Arf6 GAP domain was deleted (ΔARF GAP) or the Arf6 GAP activity was abrogated by mutagenesis (R53Q, a mutant described previously [23]) (Schematic, Fig 6A). Removal of the entire Arf6 GAP domain led to the appearance of largely immobile intracellular vesicles as analyzed by real-time fluorescence microscopy (Fig 6B, S10 and S11 Movies) and inhibited the ADAP2-mediated enhancement of macropinocytosis (Fig 6C). In addition, an Arf6 GAP activity mutant of ADAP2 (R53Q) did not induce cytoplasmic vesicles and exhibited a strong association with the cell periphery (Fig 6B, S12 Movie) and did not induce macropinocytosis (Fig 6C).

Because we found that the Arf6 GAP activity of ADAP2 was required for its induction of macropinocytosis, we next assessed whether expression of Arf6 would impact ADAP2-mediated vesicle induction and whether the Arf6 GAP activity of ADAP2 was required for its vesicle induction. We found that expression of wild-type ADAP2 with wild-type Arf6 enhanced the formation of ADAP2-containing vesicles, although these vesicles were devoid of Arf6 (S2A and S2B Fig).

### The Arf6 GAP activity of ADAP2 is required for its restriction of DENV replication

As the Arf6 GAP activity of ADAP2 was required for its induction of macropinocytosis, we next assessed whether this activity was also required for its restriction of DENV and VSV replication. We found that whereas expression of wild-type V5- or GFP-fused ADAP2 restricted DENV replication, expression of either the R53Q or ΔARF GAP mutants of ADAP2 had no significant effect on viral replication (Fig 6D). Similarly, we found that expression of R53Q-ADAP2 had no effect on VSV infection (Fig 6E). In both cases, transfection efficiency was verified by RT-qPCR (S3A–S3C Fig). Taken together, these data implicate ADAP2 in the induction of marcopinocytosis and restriction of DENV and VSV replication via its Arf6 GAP activity.

### ADAP2 inhibits DENV and VSV entry

**Fig 7:**
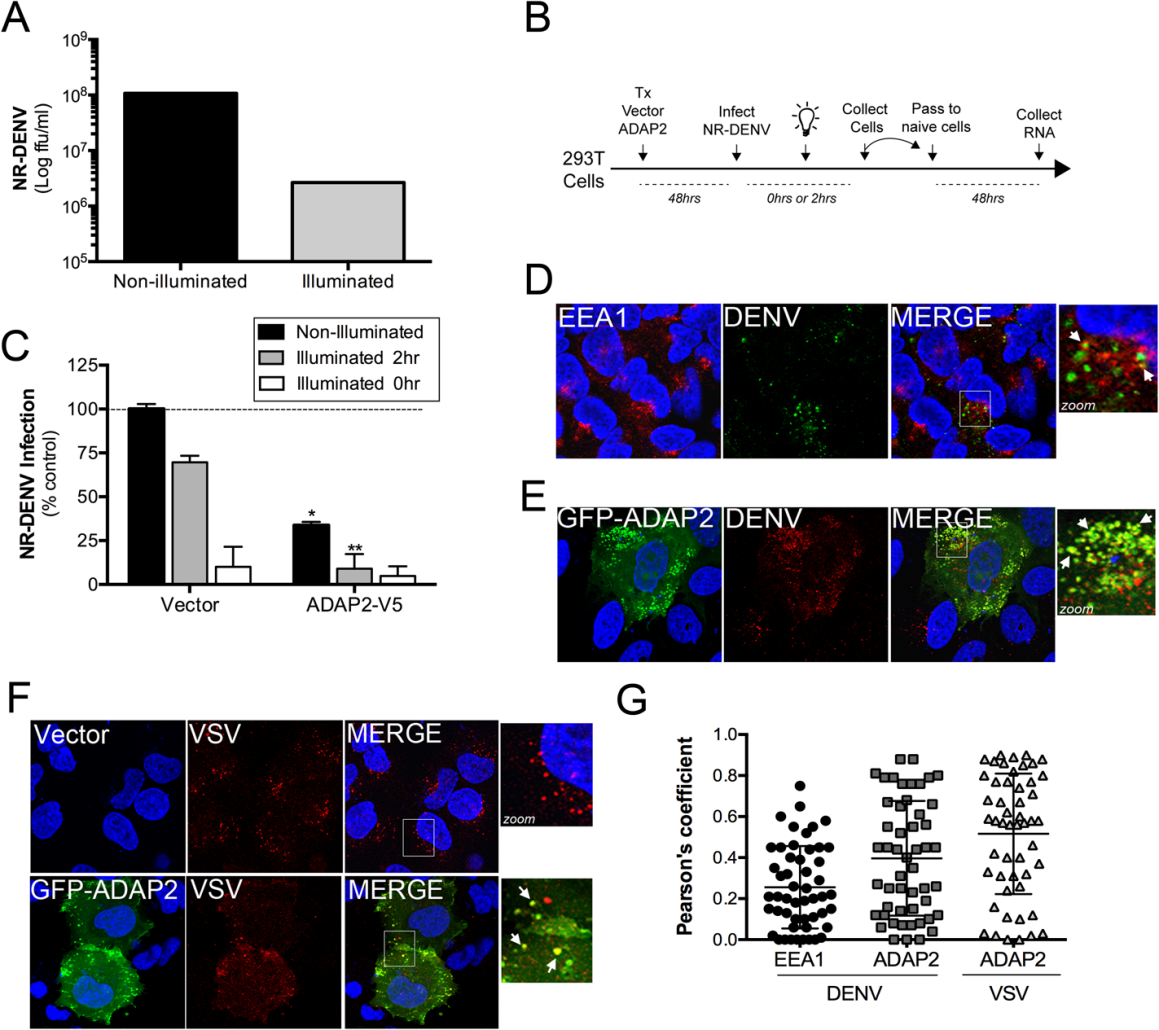
ADAP2 restricts DENV entry. **(A),**Titer of NR-DENV (shown as log FFU/mL) under non-illuminated or illuminated (at 0hr) conditions. **(B),** Schematic of experiments measuring DENV RNA release using NR-DENV. **(C),** NR-DENV infection as assessed by RT-qPCR in 293T cells transfected with vector control or ADAP2-V5 under non-illuminated (dark grey) or illuminated at 2hr (light grey) or 0hr (white) post-infection. **(D),** Confocal micrographs from U2OS cells transfected with vector control (for~48hrs) at 30min following the initiation of DENV entry. Cells were fixed and immunostained with antibodies against EEA1 (in red) and DENV (in green). DAPI-stained nuclei are shown in blue. White box denotes zoomed area shown at right. White arrows denote areas of colocalization. **(E),** Confocal micrographs from U2OS cells transfected with EGFP-ADAP2 (for~48hrs) at 30min following the initiation of DENV entry. Cells were fixed and immunostained with antibodies against DENV (in red). DAPI-stained nuclei are shown in blue. White box denotes zoomed area shown at right. White arrows denote areas of colocalization. **(F),** Confocal micrographs from U2OS cells transfected with vector control or EGFP-fused ADAP2 (for~48hrs) at 30min following the initiation of VSV entry. Cells were fixed and immunostained with antibodies against VSV-G (in red). DAPI-stained nuclei are shown in blue. White box denotes zoomed area shown at right. White arrows denote areas of colocalization. **(G),** Pearson correlation coefficient to assess the colocalization between DENV and EEA1 or ADAP2 and VSV and ADAP2, as indicated Each point represents a unique cell (>20 cells from three independent experiments). Line indicates the mean. Data in (A) and (C) are shown as mean ± standard deviation. *p<0.05 and **p<0.01.

DENV gains entry into mammalian cells via a clathrin-mediated pathway that delivers incoming viral particles to late endosomes [16, 18]. Likewise, VSV also enters cells via a clathrin-mediated pathway [10] and undergoes uncoating in early endosomes [32]. We found that expression of ADAP2 induced macropinocytosis through its Arf6 GAP activity, which was also required to restrict DENV and VSV replication. Given that DENV enters cells through a clathrin pathway, we next determined whether ADAP2 expression would alter clathrin-mediated endocytosis and/or DENV and VSV entry. We found that expression of wild-type ADAP2, but not R53Q ADAP2, altered the internalization of transferrin and prevented its perinuclear accumulation (S4A and S4B Fig), suggesting that it alters cargo internalizing via the clathrin pathway. To determine if ADAP2 restricts DENV infection at the stage of viral entry, we first generated neutral red (NR) containing DENV particles (DENV-NR). NR is an RNA-binding dye that has been used extensively in the field of picornavirus entry to identify inhibitors of virus entry [33–37], but has not been used previously in the field of flavivirus entry. When viruses are propagated in the presence of NR, the dye associates with vRNA and renders the resulting NR-containing viral particles sensitive to light. Upon virus entry and RNA release, the NR dye diffuses away from the vRNA and replication continues in a light-insensitive manner. Thus, this method is a useful tool to establish the kinetics of viral entry and/or identify agents that inhibit and/or alter the release of vRNA. By propagating DENV in the presence of NR, we successfully generated light sensitive viral particles as confirmed by a greater than two-log drop in titer when fluorescent focus forming assays were performed under illuminated conditions (Fig 7A).

To determine whether ADAP2 restricts DENV entry, we performed a modified neutral-red infectious center (NRIC) assay [33] using DENV-NR particles (a schematic of this assay is shown in Fig 7B). If ADAP2 restricts DENV entry, we would expect that exposure of ADAP2-expressing cells to light early in infection (2hrs p.i.), would elicit a greater inhibition of DENV infection than when cells are infected under non-illuminated conditions. Using this assay, we found that expression of ADAP2 inhibited DENV-NR replication under both non-illuminated and illuminated (at 2hrs p.i.) conditions (Fig 7C). However, importantly, we found that the restriction of DENV-NR replication by ADAP2 was significantly enhanced under the illuminated (at 2hrs p.i.) condition (Fig 7C), supporting a role for ADAP2 in the restriction of DENV entry.

Next, we assessed whether incoming DENV and VSV associated with ADAP2 during their entry, as would be expected if it directly impacted their entry/trafficking. Similar to a previous study [18], we found that DENV associated with early endosomes (as assessed by its association with early endosome antigen-1 (EEA1)) early in its entry (Fig 7D and 7G). However, we found that when ADAP2 was expressed, it exhibited a strong association with DENV particles early in its entry (<30min p.i.) (Fig 7E and 7G). Similar to our findings with DENV, we found that ADAP2 also associated with VSV during its entry (Fig 7F and 7G). As we have shown that ADAP2 does not associate with early endosomes, these DENV and VSV vesicles likely represent macropinosomes and/or lysosomes and suggest that ADAP2 alters the endocytic uptake of viral particles.

## Discussion

Inhibition of viral entry by ISGs is an effective strategy utilized by host cells to inhibit viral infection at the earliest stages of the viral life cycle. Here we show that ADAP2 expression is induced by type I IFNs in a STAT1-dependent manner and restricts DENV and VSV replication using an Arf6 GAP-mediated pathway, likely via the induction of macropinocytosis. Thus, our results point to a previously uncharacterized ISG that restricts DENV and VSV entry to limit viral replication.

ADAP2 is ubiquitously expressed, with the highest levels of expression in the fat, heart, and skeletal muscle [38, 39]. Unlike ADAP2, the expression of the related ADAP1 (also known as centaurin-α_1_) is largely restricted to the brain [25, 40, 41]. Although ADAP2 and ADAP1 share ~60% sequence identity and contain N-terminal ARF GAP domains and two PH domains, ADAP1 contains a nuclear localization signal and localizes primarily to the cytosol or nucleus whereas ADAP2 localizes predominantly to membrane ruffles. Sequence analysis of the promoter sequences of ADAP2 and ADAP1 revealed the presence of a conserved IFN-stimulated response element (ISRE) in the promoter of ADAP2, but not in the promoter of ADAP1 (S5 Fig)_._ The induction of genes in response to type I IFNs is controlled by the presence of a conserved promoter sequence (GAAA(N)GAAA, where N is any nucleotide) termed the ISRE. Indeed, ADAP2 has been shown to be upregulated by IFN treatment in various human cells, such as primary hepatocytes [42] and PBMCs isolated from multiple sclerosis patients undergoing IFN treatments [43]. Taken together, our findings thus suggest that ADAP2 is specifically upregulated in response to type I IFN signaling, likely due to the presence of an ISRE.

Our data suggest that ADAP2 inhibits DENV and VSV replication at the stage of viral entry. DENV enters mammalian cells by a clathrin-mediated endocytic pathway that delivers incoming viral particles to Rab7 positive late endosomes [16, 18, 44]. Although a small portion (<20%) of viral particles undergo membrane fusion in Rab5 early endosomes or Rab5/Rab7 intermediate endosomes, the majority (>80%) of membrane fusion occurs in Rab7 late endosomal compartments [35]. Thus, the delivery of incoming DENV particles to a late endosomal compartment is required for efficient membrane fusion and perturbation of this pathway could dramatically impact subsequent viral replication. Similarly, VSV enters cells via a clathrin-dependent pathway and requires Rab5-mediated delivery to an endosomal compartment (with a pH of 6.2) to trigger G-protein-mediated fusion [11, 45, 46], which is required to deliver particles into the host cell cytoplasm [32]. We show that expression of ADAP2 robustly induces macropinocytosis and that ADAP2-positive vesicles associate with Rab8a and the lysosomal marker LAMP1, but not Rab7 or Rab5. Our results therefore suggest that the induction of ADAP2 would serve to enhance macropinocytosis in cells exposed to type I IFN and that this would alter the trafficking of incoming DENV and VSV particles to preclude their delivery to Rab7 late endosomes. Moreover, the association of ADAP2-positive vesicles with LAMP1-positive compartments would serve to deliver any cargo, including viruses, contained within these vesicles to the degradative environment of lysosomes.

Similar to our findings with DENV, we found that expression of ADAP2 partially restricted VSV infection. In contrast, ADAP2 expression had no effect on CVB or SeV replication. Unlike both DENV and VSV, CVB enters nonpolarized cells via a clathrin-independent pathway and does not require specific delivery to endosomal compartments for uncoating [14]. In addition, SeV fusion occurs at the host cell surface and does not rely on specific delivery to endosomal compartments for its entry. Thus, the differential effects of ADAP2 on viruses that require delivery to an endosomal compartment (DENV and VSV) and those that do not (CVB, SeV) support a role for ADAP2 in the mislocalizing of incoming virions to non-endosomal compartments, thus preventing their uncoating/fusion. This is supported by our findings that ADAP2 expression also led to the mislocalization of transferrin, which is specifically internalized by a clathrin-mediated pathway and delivered to the endosomal network. Consistent with this, we also found that expression of wild-type or Q67L Rab8a and wild-type or Q111L Rab34, all of which induce macropinocytosis, restrict DENV infection (S6 Fig).

We found that both the anti-DENV and VSV and macropinocytosis-inducing effects of ADAP2 required its Arf6 GAP activity. The cycling of Arf6 between GTP and GDP bound states is a primary determinant for its impact on actin cytoskeletal dynamics and endosomal recycling. For example, expression of a constitutively active Arf6 mutant (Q67L) induces the accumulation of clathrin cargo in intracellular endosomal compartments, likely due to alterations in endosomal fusion [47, 48]. In contrast, expression of a dominant inactive mutant (T157N) has no effect on intracellular accumulation of cargo, but instead exhibited overall lower levels of signal, presumably due to increases in endosomal recycling [49]. These data suggest that in cells expressing ADAP2, Arf6 is maintained in a GDP-bound state, leading to both increases in macropinocytosis and endosomal recycling. Collectively, the promotion of these pathways would induce dramatic alterations in the uptake and intracellular trafficking of viral particles. Consistent with this, we found that expression of wild-type Arf6 had no effect on DENV infection whereas expression of Q67L Arf6, which dramatically enhances vesicle invaginations from the plasma membrane in actin-coated vesicles [50], potently restricted infection (S6 Fig). In some scenarios, the maintenance of Arf6 in a GDP bound state may serve in a proviral, or promicrobial, manner. Recently, several Arf GAP domain-containing molecules, including ADAP1, were shown to directly facilitate the uptake of *Salmonella* into host cell via their effects on the actin cytoskeleton [51].

Our findings presented here implicate ADAP2 as an ISG specifically induced to alter host cell endocytic and intracellular trafficking pathways to restrict viral entry. We show that the expression of ADAP2 dramatically induces macropinocytosis via an Arf6 GAP-dependent pathway, which correlates to alterations in the uptake of transferrin and in an inhibition of DENV and VSV entry and/or intracellular trafficking.

## Materials and Methods

### Cells and viruses

U2OS, 293T, Vero, 2fTGH (STAT1 wild-type) and U3A (STAT1 mutant) fibrosarcoma cells (described previously [52]) were grown in DMEM-H supplemented by 10% FBS and penicillin/streptomycin. HeLa (CCL-2) cells were grown in MEM supplemented by 5% FBS and penicillin/streptomycin. HEK293T knockout MAVS were generated as follows: cells were plated at a density of 2×10^4^ cells per well in a 96-well plate. The next day, CRISPR plasmids were transfected using GeneJuice transfection reagent (Merk Millipore) according to the manufacturer’s protocol. pRZ-mCherry-Cas9 and pLenti-gRNA constructs were transfected at a ratio of 3:1 (i.e. 150 ng: 50 ng). Critical exons of MAVS were targeted using a gRNA construct (sequence available by request). Subsequently, limiting dilution cloning was performed and after 10 days, growing monoclones were selected by bright field microscopy and positive clones trypsinized and expanded in two separate wells. One well was used to recover gDNA as previously described (Ablasser et al., 2013) and subsequently the target region of interest was amplified in a two-step PCR and subjected to deep sequencing. Knockout cell clones were identified as cell clones harboring all-allelic frame shift mutations using OutKnocker (Schmid-Burgk et al., 2014). Genotype of the respective knockout cell line is available upon request.

Experiments were performed with DENV-2 (16681) obtained from BEI Resources and expanded in C6/36 mosquito midgut cells as described previously [53] and titer was determined in Vero cells by a fluorescent foci forming unit (FFU) assay, as previously described [54]. Vesicular stomatitis virus (VSV) expressing GFP and coxsackievirus B3-RD (CVB3-RD) have been described previously [55]. Sendai virus (SeV) was purchased from Charles River Laboratories. Experiments measuring productive virus infection were performed approximately 48h post-transfection, at which time cells were infected with DENV-2 at a multiplicity of infection (MOI) of 1 FFU/cell for 24h, VSV and CVB3-RD at MOIs of 0.2 PFU/cell for 8h, SeV at 100 hemagglutination units (HAU)/mL, unless otherwise stated.

### Plasmids, siRNAs, and transfections

ADAP2-V5 and GFP-ADAP2 were constructed by amplification of human ADAP2 cDNA (clone Id: 5214358, Thermo Scientific) and cloning into pcDNA 3.1/V5-His TOPO TA Expression Kit or NT-GFP Fusion TOPO TA according to the manufacturer’s protocol (Invitrogen). DsRed-fused ADAP2 was constructed by amplification of ADAP2 cDNA followed by insertion into the XhoI and EcoRI sites of pDsRed2-C1 (Clontech). GFP-ADAP2-ΔArfGAP and GFP-ADAP2-ΔPH2 were constructed by amplification of ADAP2 cDNA with primers beginning at residue 132 or ending at residue 254, respectively, followed by cloning into NT-GFP Fusion TOPO TA according to the manufacturer’s protocol (Invitrogen). Mutagenesis was performed using Quikchange (Stratagene) according to the manufacturer’s protocol. RFP-LAMP1 (plasmid #1817), RFP-EEA1 (plasmid #42635), GFP-Rab8a (plasmid #24898), GFP-Rab8a[Q67L] (plasmid #24900), and pcDNA-HA-Arf6 (plasmid # 10834) were obtained from Addgene. EGFP-tagged Rab34 and Rab7 constructs have been described previously [56]. YFP-Actin was kindly provided by Jeffrey Bergelson, Children’s Hospital of Philadelphia.

Control (scrambled) siRNA and siRNA targeting ADAP2 (5’-GGACUGGUUCAAUGCCCUC-3’) were purchased from Sigma.

Transfection of U2OS and 293T cells with plasmids was performed using X-tremegene 9 DNA (Roche) or X-tremegene HP DNA (Roche) transfection reagents according to the manufacturer’s protocol. SiRNAs were transfected using Dharmfect-1 according to the manufacturer’s protocol. Cells were infected and/or fixed 48h post-transfection.

### Antibodies

Rabbit anti-MAVS antibody was obtained from Bethyl Laboratories. Mouse anti-HA antibody, mouse anti-V5, goat anti-EEA1 and rabbit anti-GAPDH HRP-conjugated antibodies were purchase from Santa Cruz Biotechnology. Mouse anti-VSVG (P5D4) and mouse anti-DENV (clone D3-2H2-9-21) antibodies were purchased from Santa Cruz Biotechnology and Millipore, respectively. Alexa Fluor 488 or 594 phalloidin and Alexa fluor-conjugated secondary antibodies were purchased from Invitrogen.

### Microarray analyses

We used high-throughput microarray analysis, performed as we previously described [57], to screen for transcriptional changes in control (2fTGH) *vs*. STAT1 signaling deficient (U3A) HT1080 cells, both treated with 100U of purified IFNβ (PBL) for 24hrs. In parallel, mock-treated 2fTGH and U3A were also included and were used to identify differentially expressed genes in IFNβ-treated cells. Briefly, the quality of all RNA samples was confirmed using an Agilent 2100 Bioanalyzer (Agilent Technologies, Santa Clara, CA) to ensure RNA integrity and quality. mRNA labeling was performed using a One-Color Low Input Quick Amp Labeling Kit (Agilent) and prepared for hybridization on SurePrint G3 Human Gene Expression 8x60K slides using the Gene Expression Hybridization Kit (both from Agilent). Slides were scanned using Agilent’s SureScan Microarray Scanner System, and data extracted using Agilent’s Feature Extraction Software (version 11.0.1.1). Microarray data were normalized using the cyclic loess normalization method [58]. The R package Limma (Linear Models for Microarray Data), which implements a moderated *t*-test, was used to identify differentially expressed mRNAs between mock- and IFNβ -treated samples [59]. Storey's *q*-value method [60], as implemented in R package *q*-value, was used to calculate the adjusted p-values in order to control the false discovery rate.

### Live-cell imaging

U2OS cells were transfected with indicated plasmids in glass-bottom 35mm dishes (MatTek). Approximately 48 h following transfection, plates were placed into a 37°C, CO_2_-controlled incubator positioned over a motorized inverted microscope to allow for long-term time-lapse imaging (VivaView FL; Olympus), and images captured every 10–15 min for ~24h, unless otherwise stated.

### Immunofluorescence microscopy

In all experiments, cells cultured in 8-well chamber slides (LabTek) were washed and fixed with 4% paraformaldehyde followed by permeabilization with 0.1% Triton X-100 in PBS. Cells were incubated with the indicated primary antibodies for 1 hr at room temperature, washed, and then incubated with secondary antibodies for 30 min at room temperature, washed, and mounted with Vectashield (Vector Laboratories) containing 4’,6-diamidino-2-phenylindole (DAPI). Images were captured using a FV1000 confocal laser scanning microscope (Olympus), analyzed using Image J/Fiji (NIH) or Imaris (Bitplane), and contrasted and merged using Photoshop (Adobe). For three-dimensional analysis, xy or yz series stacks were acquired at ~0.5 μm intervals through the total thickness of the cell monolayer (~10 μm). Single particle tracking was performed using the MTrackJ plugin in Image J/Fiji. For imaging quantification, >20 individual organelles from at least three unique fields were measured using Imaris. For measurements of colocalization, >20 individual cells from at least three experiments were used to calculate Pearson’s colocalization coefficients using the Coloc2 plugin in ImageJ/Fiji.

For EIPA treatment studies, U2OS cells were transfected with DsRed-ADAP2 for 48h, then treated with EIPA (102 μM, from Sigma) for 60 min in complete medium, followed by fixation/permeabilization as described above.

For studies related to viral entry, U2OS cells were transfected with vector or ADAP2 plasmids, as indicated, for ~48hrs. At this time, virus (~35 FFU/cell for DENV and ~100 PFU/cell for VSV) was preadsorbed to cells for 60mins at 16°C. Following this incubation and a brief washing to remove unbound virus, viral entry was initiated by shifting the temperature to 37°C. At the indicated times (~30min for DENV and VSV), cells were fixed in 4% PFA and immunostained as described above.

### RT-qPCR

Total cellular RNA was extracted using TRI reagent (MRC) or a GenElute total RNA miniprep kit (Sigma) according to the manufacturer’s protocol. RNA samples were treated with RNase-free DNase (Qiagen or Sigma) prior to cDNA synthesis. Total RNA(1 μg) was reverse transcribed by using iScript cDNA synthesis kit (Bio-Rad). RT-qPCR was performed using iQ SYBR green supermix (Bio-Rad) in an Applied Biosystems StepOnePlus real-time PCR machine. Gene expression was calculated using the 2^-△△CT^ method[61], normalized to actin. QuantiTect primers against ADAP2, IFI44L, DENV, VSV, were purchased from Sigma. Primer sequences were as follows: ADAP2 (5’-AAGCTGTCATCAGCATTAAG-3’ and 5’-ACTATCTCCTTCCCACTTTC-3’); IFI44L (5’-ACTAAAGTGGATGATTGCAG-3’ and 5’-TGCAGAGAGGATGAGAATATC-3’); DENV (5’-AGTTGTTAGTCTACGTGGACCGA-3’ and 5’-CGCGTTTCAGCATATTGAAAG-3’). Actin, VSV, CVB, ISG56, ISG60 and SeV primer sequences have been described [36, 62].

### Dextran uptake assay

U2OS cells were transfected as described above. Approximately 48h following transfection, cells were washed with PBS and incubated with complete medium containing 70,000-MW dextran conjugated to Oregon green 488 (0.1 mg/ml; Invitrogen) or 10,000-MW dextran conjugated to Alexa Fluor 594, as indicated, for 45 min followed by fixation/permeabilization as described above.

### Neutral red (NR)-labeled DENV

For generation of NR-DENV, C6/36 cells were infected with DENV-2 at 33°C for 1 h in FBS-free DMEM (DMEM-0) then incubated with DMEM containing 2% FBS (DMEM-2) containing 100 μg neutral red dye (Sigma) for 5 days in the dark. Following this incubation, the medium was harvested, cleared from cellular debris by low-speed centrifugation, aliquoted, and stored at −80°C. NR-DENV titers were measured in either dark or light-exposed conditions in Vero cells using a foci forming unit assay as described [54]. All experiments with NR-DENV were performed under semidark conditions, unless otherwise stated.

For experiments measuring NR-DENV infection, 293T cells were transfected with the indicated plasmids and ~ 48h post-transfection, cells were infected with NR-DENV (MOI = 5) for 2 h in the dark. At this time, cells were illuminated on a light box for 20 min. In parallel, monolayers were maintained in the dark to control for effects unrelated to entry or illuminated at 0hr post-infection to verify the light sensitivity of NR-DENV. Cells were then washed, collected by manual pipetting, and then transferred onto naive 293T cells. Cells were infected for approximately 48h, washed and infection levels assessed by RT-qPCR.

### Immunoblots

Cells were grown in 24-well plates and lysed in RIPA buffer [50 mM Tris-HCl (pH 7.4); 1% NP-40; 0.25% sodium deoxycholate; 150 mM NaCl; 1 mM EDTA; 1 mM phenylmethanesulfonyl fluoride; 1 mg/ml aprotinin, leupeptin, and pepstatin; 1 mM sodium orthovanadate]. Lysates were sonicated and insoluble material was cleared by centrifugation. Protein concentration of lysates was determined by BCA protein assay (Thermo Scientific). Lysates containing equal amounts of protein were loaded onto 4 to 20% Tris-HCl gels (Bio-Rad) and transferred to nitrocellulose membranes. Membranes were blocked in 5% nonfat dry milk, probed with the indicated antibodies, and developed with horseradish peroxidase-conjugated secondary antibodies (Santa Cruz Biotechnology), and SuperSignal West Pico or Dura chemiluminescent substrates (Pierce Bio-technology).

### Statistical analysis

Data are presented as mean ± SD unless otherwise stated, and were analyzed with Prism software (Graphpad) by two-tailed unpaired Student’s t-test. A p value <0.05 was considered significant.

## Supporting Information

S1 Table List of genes from microarray analyses in control (TGH) HT1080 or STAT1-signalinf deficient (U3A) HT1080 cells exposed to 100U of purified IFNβ for ~24hrs. Expression is shown as fold change relative to untreated (CON) cells. Gene names and accession numbers are shown at right.
(XLSX)

S1 Fig (A), Level of ADAP2 expression in 293T cells transfected with vector control or V5-ADAP2. Shown is a representative graph from the pooled data shown in Fig 1C. (B), Infection of DENV as assessed by RT-qPCR in 293T cells transfected with ADAP2-V5 or vector control and infected with the indicated MOI of DENV. Data are normalized to vector control-infected cells. (C), Infection of DENV (1 FFU/cell for 24hrs) and VSV (0.2 PFU/cell for 8hrs) as assessed by RT-qPCR in 293T cells transfected with vector control, or ADAP2-V5 or GFP-ADAP2. Data are normalized to vector control-infected cells. (D), Level of ADAP2 expression in 293T cells transfected with vector control, ADAP2-V5, or GFP-ADAP2. Shown is a representative graph from the pooled data shown in panel (C). (E), Level of ADAP2 expression in wild-type or MAVS^-/-^ 293T cells transfected with vector control of V5-ADAP2. Shown is a representative graph from the pooled data shown in Fig 1D. (F), Induction of ISG56 and IFI44L (left y-axis) in 293T cells infected with DENV (1 FFU/cell) for 24hrs that had been transfected with vector control, ADAP2-V5, or GFP-ADAP2. Data are presented as a fold ISG induction (as assessed by RT-qPCR) compared to uninfected control cells. DENV replication (right y-axis) in 293T cells infected with DENV (1 FFU/cell) for 24hrs that had been transfected with vector control, ADAP2-V5, or GFP-ADAP2. (G), Level of ADAP2 expression in 293T cells transfected with vector control, ADAP2-V5, or GFP-ADAP2. Shown is a representative graph from the pooled data shown in panel F. (H), HeLa cells were transfected with control (scrambled) siRNA CONsi) or ADAP2 siRNA (ADAP2si) for ~48hrs and then infected with DENV (0.3 FFU/cell) for 24hrs. Infection (left y-axis) and level of ADAP2 expression (right y-axis) were measured by RT-qPCR and normalized to CONsi-transfected cells. In all, data in are shown as mean ± standard deviation. *p<0.05, **p<0.01, ***p<0.001.
(TIF)

S2 Fig (A), Confocal micrographs of U2OS cells transfected with wild-type, R53Q, or ΔArf GAP GFP-fused ADAP2 and wild-type Arf6. (B), Quantification of the numbers of ADAP2+ vesicles in cells transfected with either wild-type ADAP2 (left) or R53Q ADAP2 (right) and either vector control or Arf6. Shown are the numbers of ADAP2+ vesicles per cell from individual cells expressing the indicated constructs. *p<0.01.
(TIF)

S3 Fig (A, B), Level of ADAP2 expression in 293T cells transfected with vector control or V5-fused wild-type or R53Q V5-ADAP2 (A) or EGFP-fused wild-type or ΔArfGAP ADAP2 (B). Shown is a representative graph from the pooled data shown in Fig 6D. (C), Level of ADAP2 expression in 293T cells transfected with vector control or V5-fused wild-type or R53Q V5-ADAP2. Shown is a representative graph from the pooled data shown in Fig 6E.
(TIF)

S4 Fig (A), U2OS cells transfected with GFP-ADAP2 were incubated with Alexa Fluor 594 transferrin (red) for 45 min at 37°C and then fixed and processed for confocal microscopy as described in Materials and Methods. DAPI-stained nucleo are shown in blue. (B), Quantification of perinuclear Alexa Fluor 594 transferrin localization in U2OS cells transfected with vector control or wild-type or R53Q GFP-ADAP2. A total of fifty cells were quantified.
(TIF)

S5 Fig Promoter sequences of ADAP2 (top) or ADAP1 (bottom). ISRE in ADAP2 is highlighted in blue.
(TIF)

S6 Fig DENV infection as assessed by RT-qPCR in 293T cells transfected with vector control (black bar) or V5-ADAP2 (grey bar), HA-Arf6 wild-type or Q67L (blue bars), EGFP-Rab8a wild-type or Q67L (orange bars), or EGFP-Rab34 wild-type of Q111L (green bars). Data are shown as percent infection relative to vector transfected controls and are shown as mean ± standard deviation (*p<0.05, ** p<0.01, ***p<0.001).
(TIF)

S1 Movie Real-time imaging of U2OS cells transfected with GFP-ADAP2. Approximately 48hrs post-transfection, images were captured every 10 minutes. Shown are movies for differential interference contrast (DIC, right) and GFP (left).
(MOV)

S2 Movie Real-time imaging of U2OS cells transfected with DsRed-ADAP2 and YFP-Actin. Approximately 48hrs post-transfection, images were captured every 10 minutes. Shown are movies for differential interference contrast (DIC, second from left), YFP (third from left), DsRed (right), and merged (left).
(MOV)

S3 Movie Real-time imaging of U2OS cells transfected with DsRed-ADAP2. Approximately 48hrs post-transfection, images were captured every 10 minutes. Shown are movies for differential interference contrast (DIC, right) and DsRed (left).
(MOV)

S4 Movie Real-time imaging of U2OS cells transfected with wild-type GFP-Rab34 and DsRed-ADAP2. Approximately 48hrs post-transfection, images were captured every 10 minutes. Shown are movies for GFP (middle), DsRed (right), and merged (left).
(MOV)

S5 Movie Real-time imaging of U2OS cells transfected with wild-type GFP-Rab8a and DsRed-ADAP2. Approximately 48hrs post-transfection, images were captured every 10 minutes. Shown are movies for GFP (middle), DsRed (right), and merged (left).
(MOV)

S6 Movie Real-time imaging of U2OS cells transfected with Q67L GFP-Rab8a and DsRed-ADAP2. Approximately 48hrs post-transfection, images were captured every 10 minutes. Shown are movies for GFP (middle), DsRed (right), and merged (left).
(MOV)

S7 Movie Real-time imaging of U2OS cells transfected with GFP-ADAP2 and mRFP-EEA1. Approximately 48hrs post-transfection, images were captured every 10 minutes.
(MOV)

S8 Movie Real-time imaging of U2OS cells transfected with GFP-Rab7 and DsRed-ADAP2. Approximately 48hrs post-transfection, images were captured every 10 minutes.
(MOV)

S9 Movie Real-time imaging of U2OS cells transfected with GFP-ADAP2 and mRFP-LAMP1. Approximately 48hrs post-transfection, images were captured every 10 minutes.
(AVI)

S10 Movie Real-time imaging of U2OS cells transfected with wild-type GFP-ADAP2. Approximately 48hrs post-transfection, images were captured every 10 minutes.
(MOV)

S11 Movie Real-time imaging of U2OS cells transfected with GFP-ADAP2 lacking the Arf GAP domain (ΔArfGAP). Approximately 48hrs post-transfection, images were captured every 10 minutes.
(MOV)

S12 Movie Real-time imaging of U2OS cells transfected with R53Q GFP-ADAP2. Approximately 48hrs post-transfection, images were captured every 10 minutes.
(MOV)
