# Supplementary figures and images for: ADAP2 Is an Interferon Stimulated Gene That Restricts RNA Virus Entry
Source: PLoS Pathog. 2015 Sep 15;11(9):e1005150. doi: 10.1371/journal.ppat.1005150 (PMC4570769; doi:10.1371/journal.ppat.1005150)

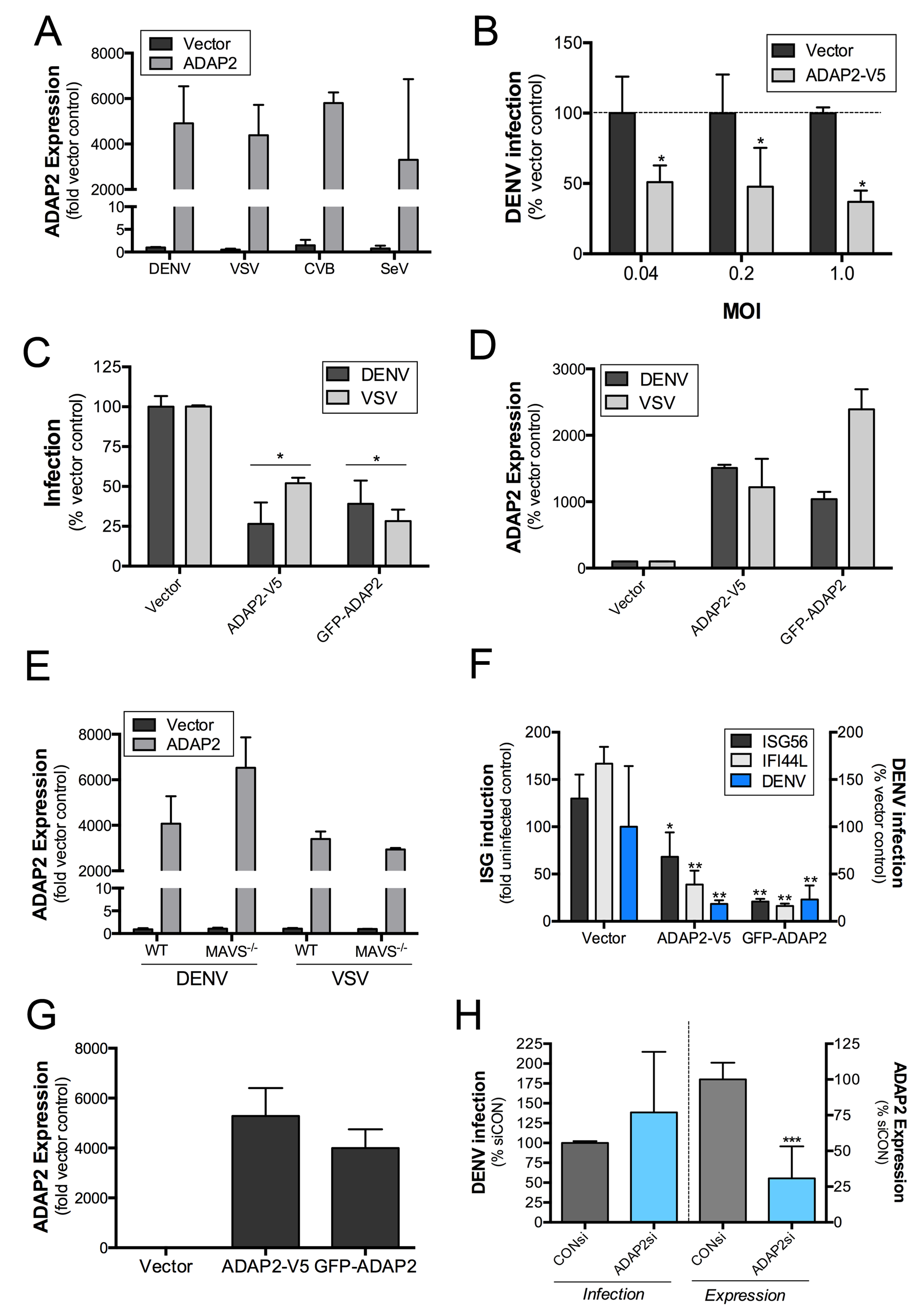

Supplement: S1 Fig — Shown is a representative graph from the pooled data shown in Fig 1C. (B), Infection of DENV as assessed by RT-qPCR in 293T cells transfected with ADAP2-V5 or vector control and infected with the indicated MOI of DENV. Data are normalized to vector control-infected cells. (C), Infection of DENV (1 FFU/cell for 24hrs) and VSV (0.2 PFU/cell for 8hrs) as assessed by RT-qPCR in 293T cells transfected with vector control, or ADAP2-V5 or GFP-ADAP2. Data are normalized to vector control-infected cells. (D), Level of ADAP2 expression in 293T cells transfected with vector control, ADAP2-V5, or GFP-ADAP2. Shown is a representative graph from the pooled data shown in panel (C). (E), Level of ADAP2 expression in wild-type or MAVS-/- 293T cells transfected with vector control of V5-ADAP2. Shown is a representative graph from the pooled data shown in Fig 1D. (F), Induction of ISG56 and IFI44L (left y-axis) in 293T cells infected with DENV (1 FFU/cell) for 24hrs that had been transfected with vector control, ADAP2-V5, or GFP-ADAP2. Data are presented as a fold ISG induction (as assessed by RT-qPCR) compared to uninfected control cells. DENV replication (right y-axis) in 293T cells infected with DENV (1 FFU/cell) for 24hrs that had been transfected with vector control, ADAP2-V5, or GFP-ADAP2. (G), Level of ADAP2 expression in 293T cells transfected with vector control, ADAP2-V5, or GFP-ADAP2. Shown is a representative graph from the pooled data shown in panel F. (H), HeLa cells were transfected with control (scrambled) siRNA CONsi) or ADAP2 siRNA (ADAP2si) for ~48hrs and then infected with DENV (0.3 FFU/cell) for 24hrs. Infection (left y-axis) and level of ADAP2 expression (right y-axis) were measured by RT-qPCR and normalized to CONsi-transfected cells. In all, data in are shown as mean ± standard deviation. *p<0.05, **p<0.01, ***p<0.001. (TIF) [file ppat.1005150.s002.tif]

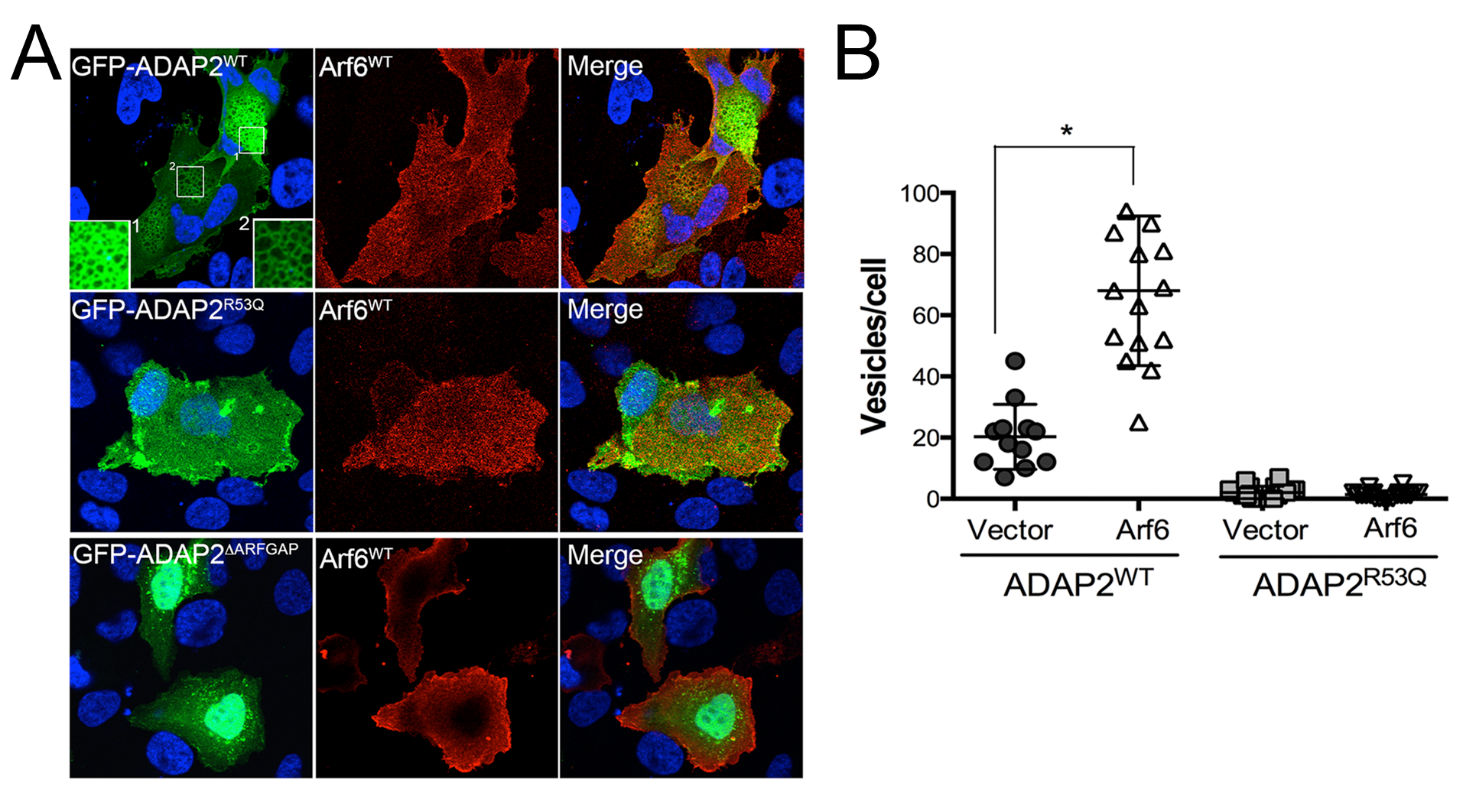

Supplement: S2 Fig — (B), Quantification of the numbers of ADAP2+ vesicles in cells transfected with either wild-type ADAP2 (left) or R53Q ADAP2 (right) and either vector control or Arf6. Shown are the numbers of ADAP2+ vesicles per cell from individual cells expressing the indicated constructs. *p<0.01. (TIF) [file ppat.1005150.s003.tif]

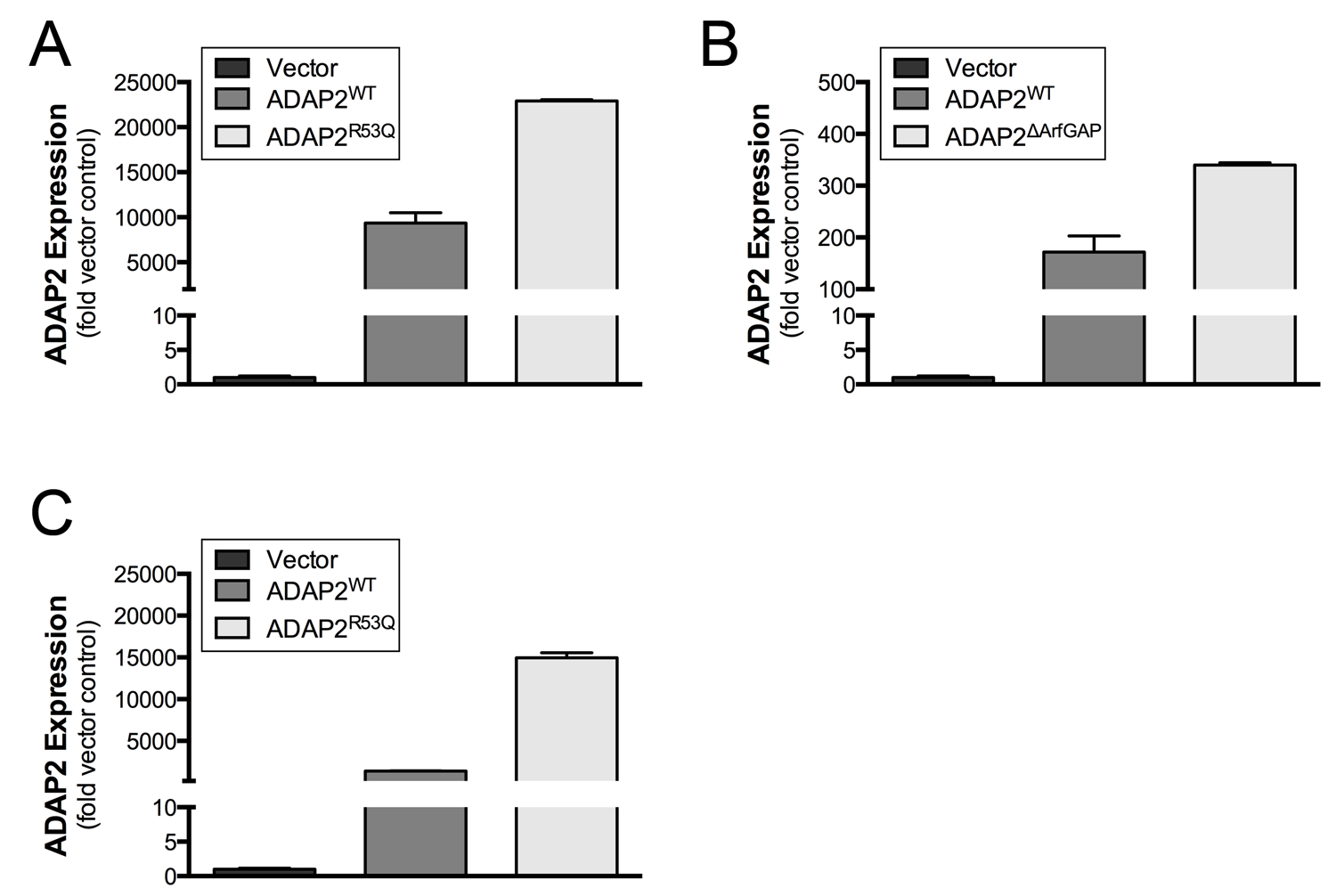

Supplement: S3 Fig — Shown is a representative graph from the pooled data shown in Fig 6D. (C), Level of ADAP2 expression in 293T cells transfected with vector control or V5-fused wild-type or R53Q V5-ADAP2. Shown is a representative graph from the pooled data shown in Fig 6E. (TIF) [file ppat.1005150.s004.tif]

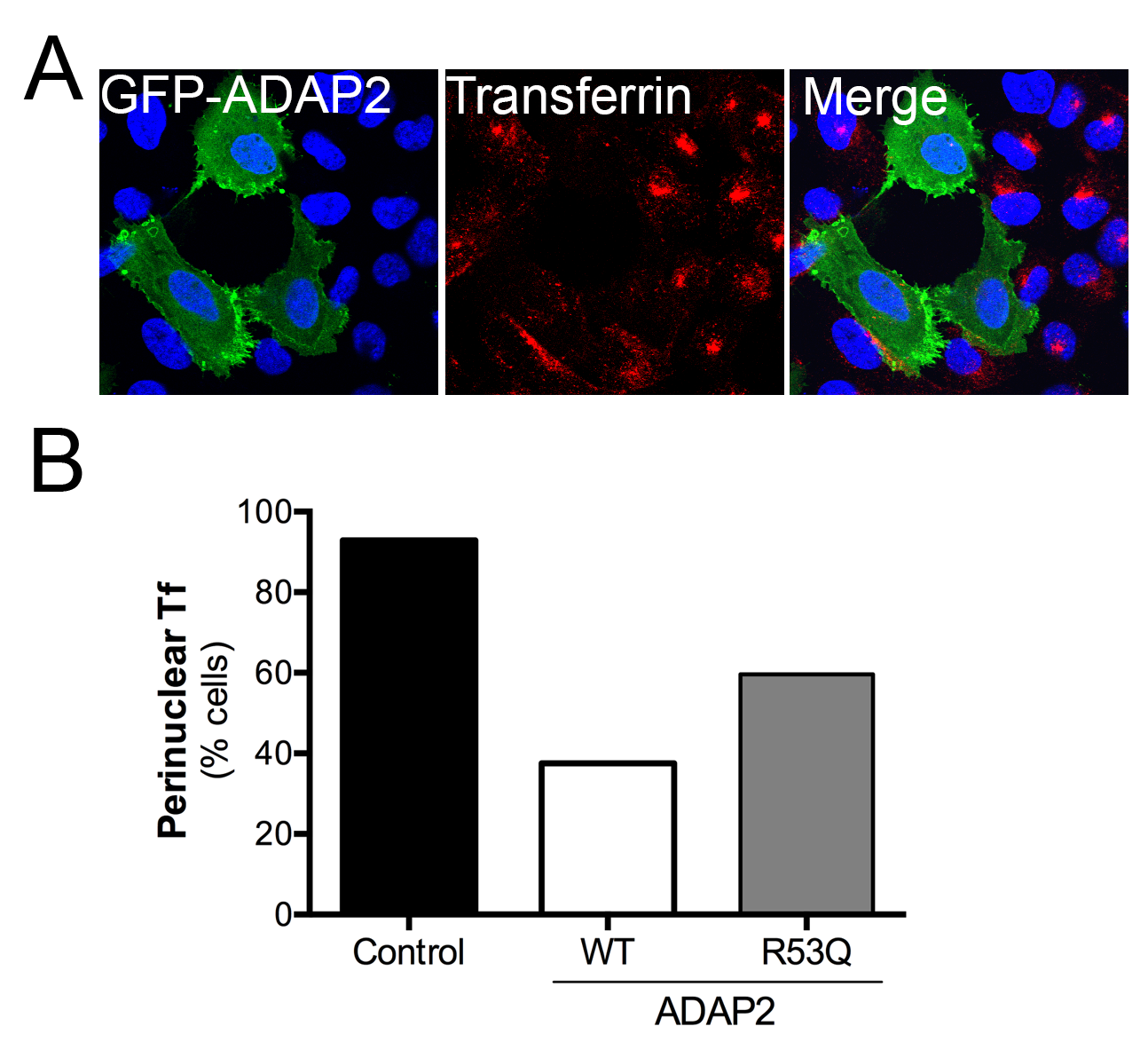

Supplement: S4 Fig — DAPI-stained nucleo are shown in blue. (B), Quantification of perinuclear Alexa Fluor 594 transferrin localization in U2OS cells transfected with vector control or wild-type or R53Q GFP-ADAP2. A total of fifty cells were quantified. (TIF) [file ppat.1005150.s005.tif]

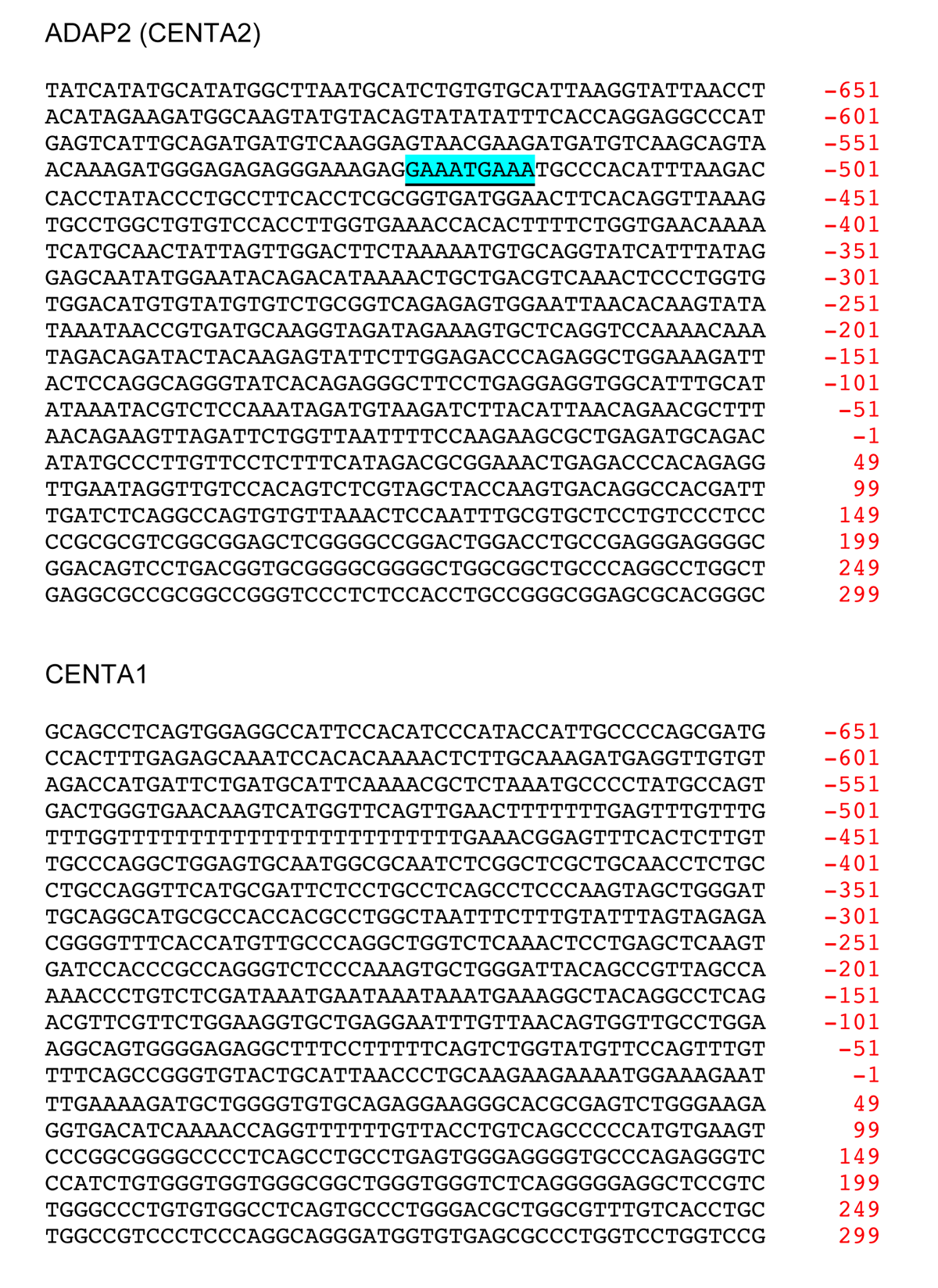

Supplement: S5 Fig — ISRE in ADAP2 is highlighted in blue. (TIF) [file ppat.1005150.s006.tif]

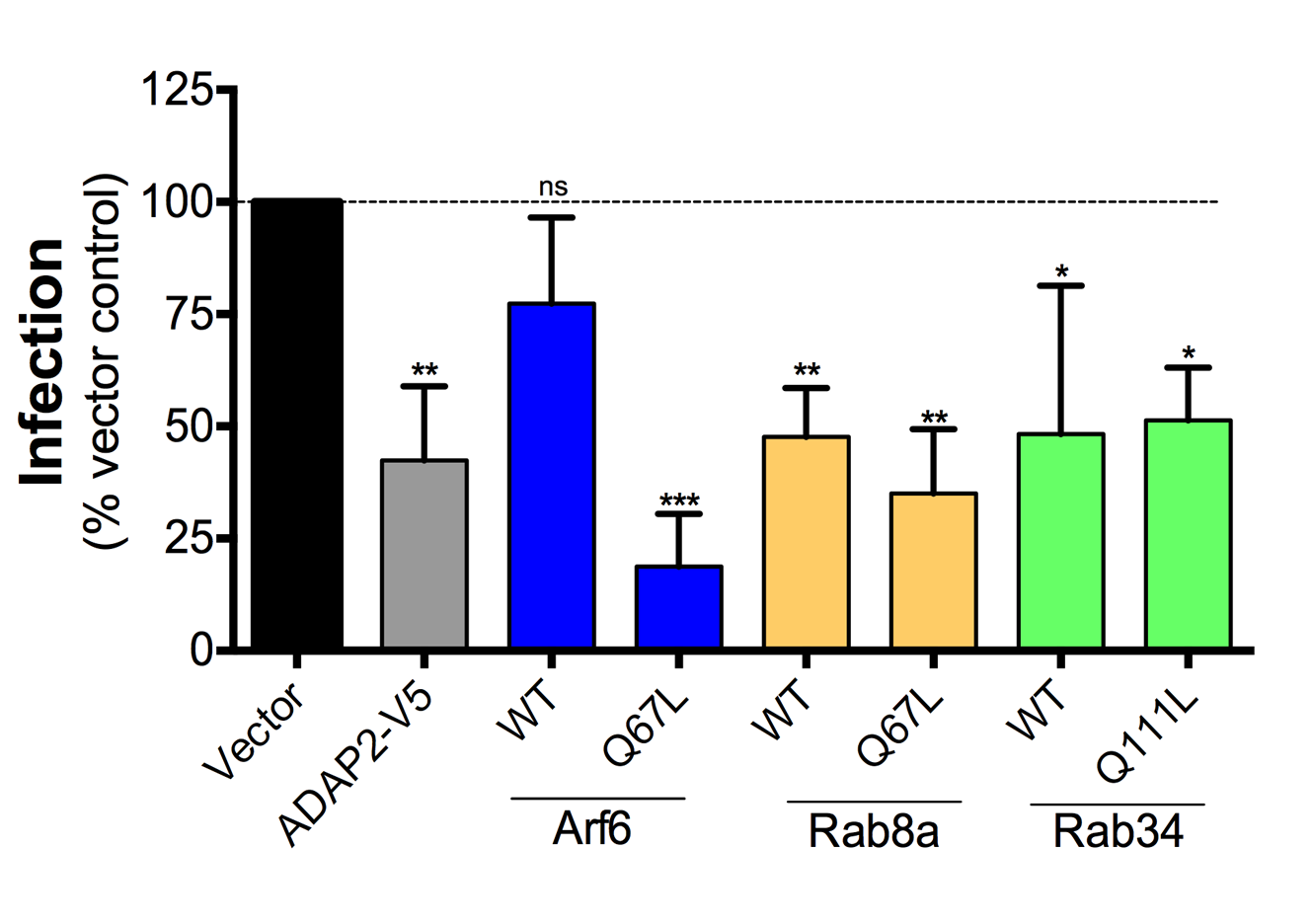

Supplement: S6 Fig — Data are shown as percent infection relative to vector transfected controls and are shown as mean ± standard deviation (*p<0.05, ** p<0.01, ***p<0.001). (TIF) [file ppat.1005150.s007.tif]
